# Supplementary material for: Hybrid Models and Biological Model Reduction with PyDSTool
Source: PLoS Comput Biol. 2012 Aug 9;8(8):e1002628. doi: 10.1371/journal.pcbi.1002628 (PMC3415397; doi:10.1371/journal.pcbi.1002628)
Supplement: Text S4 — Complete source code for the PyDSTool package (version 0.88.120504). Includes API documentation and help files linking to web pages. This file is identical to the current public release on Sourceforge.net. (ZIP) [file pcbi.1002628.s004.zip › PyDSTool/html/PyDSTool.MProject.GenTransform-class.html]

xml version="1.0" encoding="ascii"?


PyDSTool.MProject.GenTransform


| Home | Trees | Indices | Help | PyDSTool |
| --- | --- | --- | --- | --- |

Package PyDSTool ::
Module MProject ::
Class GenTransform

[hide private]

[frames] | no frames]

# Class GenTransform

source code

```
object --+
         |
        GenTransform
```

---

Generator Transformer class. Acts on GDescriptor objects that define
Generators. For these, the only non-trivial transformations are inside
the modelspec attribute.


|  |  |
| --- | --- |
| Instance Methods | [hide private] |

|  |  |
| --- | --- |
| \_\_init\_\_(self, name, gen, model\_icvalues=None, model\_parvalues=None, model\_inputs=None)  x.\_\_init\_\_(...) initializes x; see x.\_\_class\_\_.\_\_doc\_\_ for signature | source code |

|  |  |
| --- | --- |
| remove(self, obj)  Remove component, parameter, variable, input, function | source code |

|  |  |
| --- | --- |
| add(self, parent\_name, obj)  Add component, parameter, variable, input, function | source code |

|  |  |
| --- | --- |
| findStaticVars(self)  Find RHSfuncSpec variables with RHS=0 | source code |

|  |  |
| --- | --- |
| changeTargetGen(self, target)  Change target generator type. | source code |

|  |  |
| --- | --- |
| changeDomain(self, obj\_name, domain)  Change valid domain of a quantity | source code |

|  |  |
| --- | --- |
| redefineQuantity(self, obj\_name, specstr)  Redefine a Quantity using a new specification string, leaving its type unchanged. | source code |

|  |  |
| --- | --- |
| convertQuantity(self, obj\_name, targetType, targetSpecType=None)  Convert quantity between parameter, variable, or input types. | source code |

|  |  |
| --- | --- |
| convertComponent(self, obj\_name, targetType)  Convert component object to given type (provide actual type), provided the new type is compatible with the old one. | source code |

|  |  |
| --- | --- |
| makeStaticVar(self, obj\_name)  Force RHSfuncSpec variable to have RHS=0. | source code |

|  |  |
| --- | --- |
| unresolved(self)  Returns the generator spec's remaining free symbols. | source code |

|  |  |
| --- | --- |
| commit(self, new\_name)  Verifies completeness of definition before returning new generator spec. | source code |

**Inherited from `object`**:
`__delattr__`,
`__getattribute__`,
`__hash__`,
`__new__`,
`__reduce__`,
`__reduce_ex__`,
`__repr__`,
`__setattr__`,
`__str__`


|  |  |
| --- | --- |
| Properties | [hide private] |

**Inherited from `object`**:
`__class__`


|  |  |
| --- | --- |
| Method Details | [hide private] |

|  |  |
| --- | --- |
| \_\_init\_\_(self, name, gen, model\_icvalues=None, model\_parvalues=None, model\_inputs=None)  *(Constructor)* | source code |

x.\_\_init\_\_(...) initializes x; see x.\_\_class\_\_.\_\_doc\_\_ for
signature

Overrides: object.\_\_init\_\_: *(inherited documentation)*

|  |  |
| --- | --- |
| changeTargetGen(self, target) | source code |

Change target generator type. Target is a string name of the Generator
class.

|  |  |
| --- | --- |
| convertQuantity(self, obj\_name, targetType, targetSpecType=None) | source code |

Convert quantity between parameter, variable, or input types. If
parameter -> variable, the RHS will be set to zero ('static'
variable).

  


| Home | Trees | Indices | Help | PyDSTool |
| --- | --- | --- | --- | --- |

|  |  |
| --- | --- |
| Generated by Epydoc 3.0.1 on Fri May 4 15:24:06 2012 | http://epydoc.sourceforge.net |
